# Prognostic impact of liver metastases on immunotherapy in patients with advanced solid tumors: A secondary analysis of MSK-IMPACT retrospective cohort

**DOI:** 10.1097/MD.0000000000046137

**Published:** 2025-11-21

**Authors:** Shan Lin, Xiaoruo Yu

**Affiliations:** a Department of Hepatobiliary Surgery, Mengchao Hepatobiliary Hospital of Fujian Medical University, Fuzhou, Fujian Province, China; b Department of Nursing, Mengchao Hepatobiliary Hospital of Fujian Medical University, Fuzhou, Fujian Province, China.

**Keywords:** advanced solid tumors, immune-checkpoint inhibitors, liver metastasis, prognosis

## Abstract

The liver is one of the common metastatic sites of advanced solid tumors, and the prognosis of patients with liver metastases treated with immune checkpoint inhibitors (ICIs) is mostly poor. In this study, we further investigated the effect of liver metastasis on the prognosis of ICIs treatment for advanced solid tumors. Patients who received at least one ICIs treatment from 2011 to 2017 were selected from Memorial Sloan Kettering Cancer Center. Univariate and multivariate analyses were performed using Cox proportional hazards models. In addition, we examined the hazard ratios of subgroups using a stratified analysis method and assessed the model’s sensitivity by propensity score. A total of 901 patients treated with ICIs were selected for secondary analysis in this study. Median overall survival was significantly shorter for liver metastases compared with patients without liver metastases (10.0 vs 25 months, *P* < .0001). Besides, liver metastasis was associated with higher mortality for those survivors ≥ 6 months (hazard ratio [HR]: 1.84; 95% confidence interval [CI]: 1.30–2.60), ≥ 1 year (HR: 1.83; 95% CI: 1.09–3.08), and ≥ 2 years (HR: 2.42; 95% CI: 1.01–5.78). In stratified analysis, male patients had a 123% increased risk of death (HR: 2.23, 95% CI: 1.65–3.00), which was significantly higher than that of female patients (HR: 1.30, 95% CI: 0.89–1.89; *P* for interaction < .048). After adjusting for potential confounders, the prognosis of patients with liver metastases remained an independent risk factor for ICIs (HR: 1.67, 95% CI: 1.31–2.12). Liver metastasis may be an independent risk factor for advanced solid tumors treated with ICIs.

## 1. Introduction

Tumor metastasis is responsible for more than 90% of deaths associated with solid malignancies and remains a critical barrier to effective cancer treatment. The liver serves as a primary site of distant metastases in advanced cancers such as colorectal carcinoma, pancreatic adenocarcinoma, melanoma, lung cancer, and breast cancer.^[1]^ Immune checkpoint inhibitors (ICIs) targeting cytotoxic T lymphocyte-associated antigen 4 (CTLA-4), programmed cell death protein 1 (PD-1), and its ligand PD-L1 have revolutionized the management of advanced malignancies, significantly improving overall survival outcomes.^[2]^

However, emerging evidence suggests limited clinical benefits of ICIs therapy in patients with liver metastases (LM).^[3–8]^ For instance, a secondary analysis of a randomized clinical trial investigating first-line pembrolizumab for mismatch repair-deficient metastatic colorectal cancer revealed markedly shorter progression-free survival in LM patients compared to those without LM, despite the drug’s overall efficacy.^[3]^

In contrast, the IMpower150 trial demonstrated that combining atezolizumab and bevacizumab with chemotherapy conferred survival benefits in LM populations.^[9,10]^ Furthermore, 2 meta-analysis studies showed that no statistically significant association of liver metastases with the efficacy of treatments with PD-1 or PD-L1 inhibitors was found in the treatment of advanced or metastatic cancer,^[11]^ including non-small cell lung cancer (NSCLC).^[12]^ To clarify the prognostic impact of LM on ICIs responsiveness and optimize clinical strategies for these patients, we conducted a retrospective secondary analysis utilizing the Memorial Sloan Kettering-Integrated Mutation Profiling of Actionable Cancer Targets (MSK-IMPACT) cohort.^[13]^

## 2. Methods and patient

### 2.1. Patient selection

**Figure 1.**
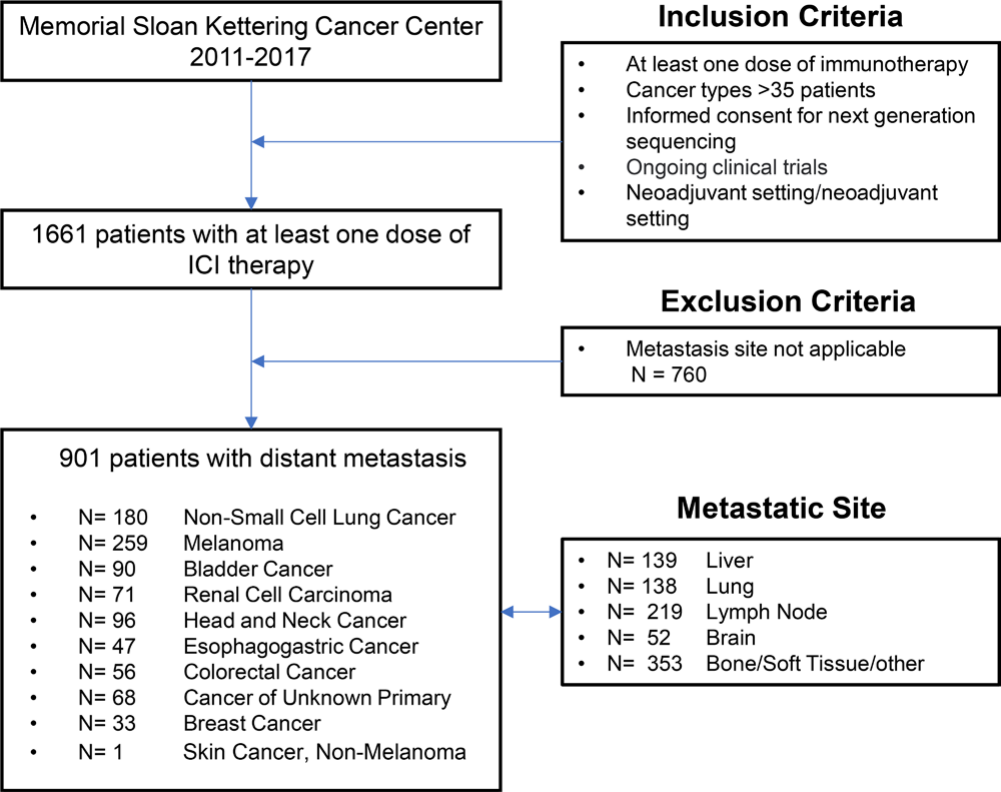
Flowchart of the study.

From 2011 until 2017, 1661 patients were collected from Memorial Sloan Kettering Cancer Center records who received at least one ICIs treatment. All patients underwent MSK-IMPACT testing in routine clinical care. Figure 1 shows the flowchart of this study, and details of tumor mutational burden measured by targeted next-generation sequencing panels have been fully described.^[13]^

The Institutional Review Board of Memorial Sloan-Kettering Cancer Center had approved the study protocol (NCT01775072). Informed consent was waived as this was a secondary analysis study.

### 2.2. Variables and definitions

Overall survival (OS) refers to the period from the first ICIs treatment to death or the last follow-up. Tumor mutation burden (TMB) is the total number of substitutions, insertions/ deletions of mutations per million bases in the coding region of exons assessed by genetic testing of tumor samples. In order to calculate TMB, this data normalizes the total number of somatic nonsynonymous mutations to the total number of megabases sequenced. Since there are no missing datasets in the source data, the processing procedures for missing data are not involved in this study. Measures included were sex, age, TMB score, cancer type, type of drug, and year of initiation of ICIs.

ICIs in this paper include atezolizumab, avelumab, durvalumab, ipilimumab, nivolumab, pembrolizumab, or tremelimumab. Advanced solid tumors included NSCLC (n = 180), melanoma (n = 260), bladder cancer (n = 90), renal cell carcinoma (n = 71), head and neck cancer (n = 96), esophagogastric cancer (n = 47), colorectal cancer (n = 56), cancer of unknown primary (n = 68), breast cancer (n = 33). Metastatic sites included the liver (n = 139), lung (n = 138), lymph nodes (n = 219), brain (n = 52), and bone/soft tissue/other (n = 352).

### 2.3. Statistical analysis

In this study, we first divided the population into 2 groups according to liver metastasis status (yes or no). In both subgroups, the Kruskal–Wallis test was used to compare continuous variables, while the Chi-square test (Fisher exact test) was used for categorical data analysis. Survival analyses were performed using the Kaplan–Meier method, and *P* values were reported with the log-rank test.

To investigate the relationship between liver metastases and OS in advanced solid tumors, univariate and multivariate analyses were performed based on Cox proportional hazards models. The results were presented as hazard ratios (HR) with 95% confidence intervals (95% CI). In addition, we examined HR using stratified analysis for subgroups of gender, age (<65, ≥ 65), TMB score (<10, ≥ 10), cancer type (NSCLC, melanoma, other), drug type (PD-1/PD-L1, Combo, CTLA-4), and ICI start year (2011–2015, 2015–2017).

### 2.4. Sensitivity analysis

Finally, we applied 3 methods to assess the core results in the sensitivity analysis. A propensity score (PS) matching analysis was first performed, with one-to-four matching completed using PS logical nearest-neighbor matching based on gender, age (<65, ≥ 65), TMB score (<10, ≥10), cancer type (NSCLC, melanoma, other), and drug type (PD-1/PD-L1, Combo, CTLA-4). The caliper width is 0.01 times the log standard deviation of PS. The C-index of the model was 0.642. Because differences in baseline characteristics remained significant after PS matching (see Table S1, Supplemental Digital Content, https://links.lww.com/MD/Q754), we also used Cox proportional hazards models adjusted for PS and eliminated inherent differences between the 2 groups by inverse probability of treatment weighted (IPTW), which was finally calculated as 1/PS for patients with liver metastases and 1/(1-PS) for patients without liver metastases.

## 3. Results

### 3.1. Descriptive characteristics

From the 2011 analysis to 2017, 1661 patients who received at least one ICIs treatment were selected for this study. Patients with metastatic sites not applicable were excluded, and a total of 901 patients were finally included in the analysis. In this study, 451 of 901 patients died, of which 89 were patients with liver metastasis, accounting for 64.03% of patients in the liver metastasis group, while 362 patients died without liver metastasis, accounting for 47.51%.

Patients were divided into 2 groups according to the presence or absence of liver metastasis, of which 139 (15.43%) patients had liver metastasis, and 762 (84.57%) patients had no liver metastasis. In Table 1, we can see that there was no significant difference in age, gender, treatment regimen, and ICIs treatment time between the 2 groups, and it was unevenly distributed in tumor type (*P < *.001) and TMB score (*P* = .022). In the liver metastasis group, the mean age of patients was 61.1 ± 13.2, the majority were male, most had TMB < 10, and the main tumor types included NSCLC, melanoma, and colorectal cancer, and PD-1/PD-L1 (70.5%) was the main treatment (Table 1).

**Table 1:** Baseline characteristics of advanced solid tumors with or without liver metastasis.

	Liver metastasis		*P*-value
	No	Yes	
N	762	139	
Age (yr)			.304
≤30	28 (3.7%)	2 (1.4%)	
31–50	124 (16.3%)	30 (21.6%)	
51–60	199 (26.1%)	30 (21.6%)	
61–70	220 (28.9%)	43 (30.9%)	
≥71	191 (25.1%)	34 (24.5%)	
Mean + SD	61.6 ± 13.9	61.1 ± 13.2	.734
Sex			.749
Male	466 (61.2%)	83 (59.7%)	
Female	296 (38.8%)	56 (40.3%)	
TMB score			.022
<10	494 (64.8%)	104 (74.8%)	
≥10	268 (35.2%)	35 (25.2%)	
Median (Min-Max)	6.9 (0.0–178.4)	5.6 (0.0–181.8)	.074
Cancer type			<.001
NSCLC	149 (19.6%)	31 (22.3%)	
Melanoma	228 (29.9%)	31 (22.3%)	
Bladder cancer	77 (10.1%)	13 (9.4%)	
Renal cell carcinoma	69 (9.1%)	2 (1.4%)	
Head and neck cancer	88 (11.5%)	8 (5.8%)	
Esophagogastric cancer	38 (5.0%)	9 (6.5%)	
Colorectal cancer	30 (3.9%)	26 (18.7%)	
Cancer of unknown primary	55 (7.2%)	13 (9.4%)	
Breast cancer	27 (3.5%)	6 (4.3%)	
Skin cancer, non-melanoma	1 (0.1%)	0 (0.0%)	
Year of ICI start			.868
2011–2012	12 (1.6%)	2 (1.4%)	
2013–2014	94 (12.3%)	15 (10.8%)	
2015–2017	656 (86.1%)	122 (87.8%)	
Drug type			.419
PD-1/PD-L1	556 (73.0%)	98 (70.5%)	
Combo	137 (18.0%)	31 (22.3%)	
CTLA-4	69 (9.1%)	10 (7.2%)	

Differences are compared using the chi-square test (or Fisher exact test) for categorical measures and the Kruskal − Wallis test for continuous measures.

CTLA-4 = cytotoxic T lymphocyte-associated antigen 4, ICI = immune checkpoint inhibitors, NSCLC = non-small cell lung cancer, PD1 = programmed cell death protein 1, TMB = tumor mutation burden.

### 3.2. Relationship between liver metastasis and overall survival

**Figure 2.**
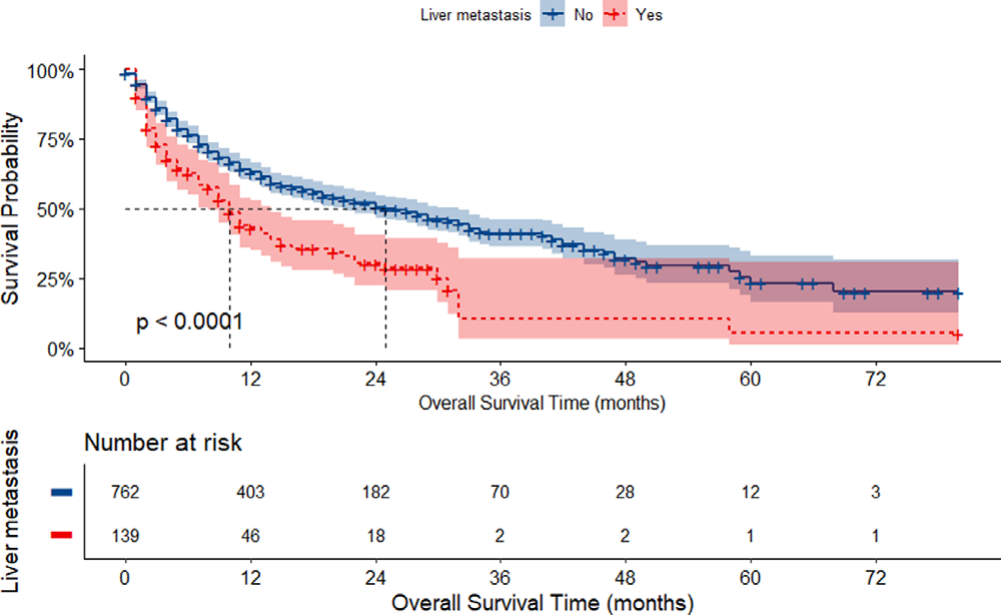
Kaplan–Meier curves for overall survival stratified by liver metastasis status in the derivation cohort.

**Figure 3.**
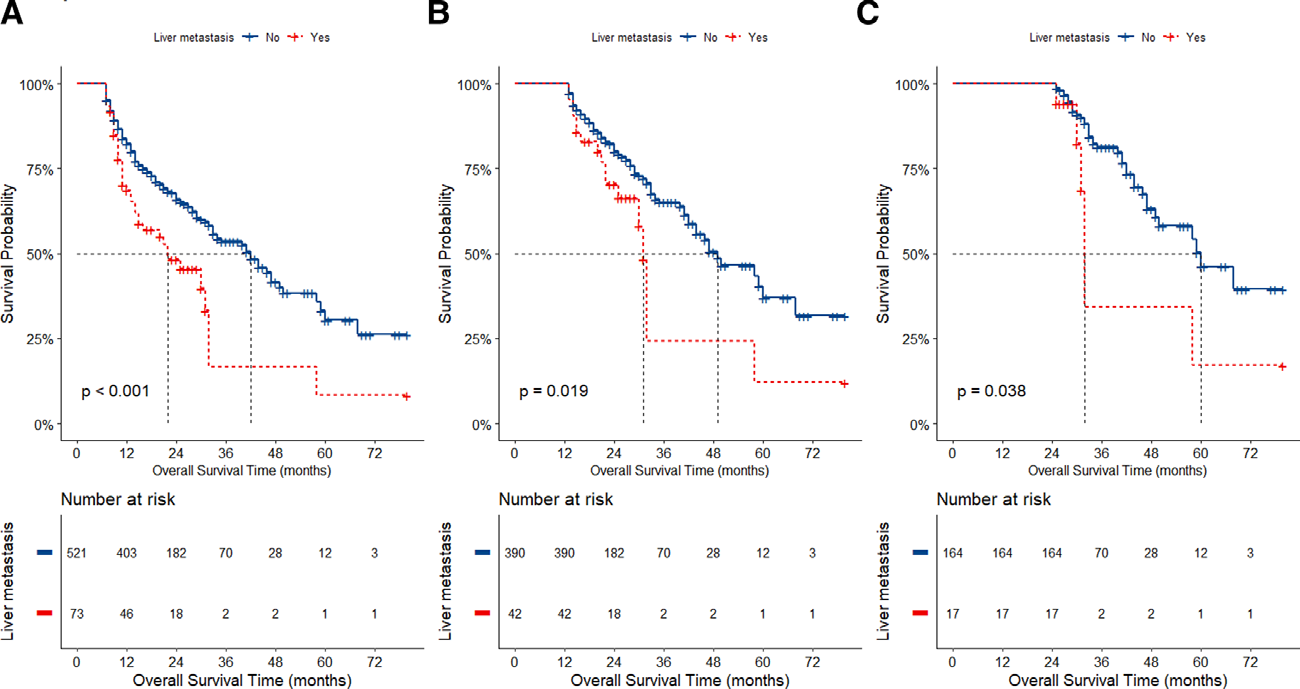
Landmark analyses of overall survival for long-term (A: ≥6 months, B: ≥1 year, and C: ≥2 years) survivors. A: HR, 1.84; 95% CI: 1.30, 2.60. B: HR,1.83; 95% CI: 1.09, 3.08. C: HR, 2.42; 95% CI: 1.01, 5.78. CI = confidence interval, HR = hazard ratio.

As shown in Figure 2, the median OS time for the 762 patients without liver metastases was 25.0 months (95% CI: 21.0–32.0) compared with 10.0 months (95% CI: 8.0–14.0) for patients with liver metastases, which was significantly lower for patients with liver metastases than for patients without liver metastases (*P < *.0001, Fig. 2). At the same time, survival was significantly shorter in the liver metastasis group compared with patients with metastases at other sites (*P < *.0001, Figure S1, Supplemental Digital Content, https://links.lww.com/MD/Q754). In the sequenced landmark analysis, Figure 3 showed that patients with liver metastasis group had significantly higher mortality than those without liver metastasis (≥6 months: HR: 1.84, 95% CI:1.30–2.60; ≥ 1 year: HR: 1.83, 95% CI: 1.09–3.08; ≥ 2 years: HR: 2.42, 95% CI: 1.01–5.78. *P < *.05).

**Figure 4.**
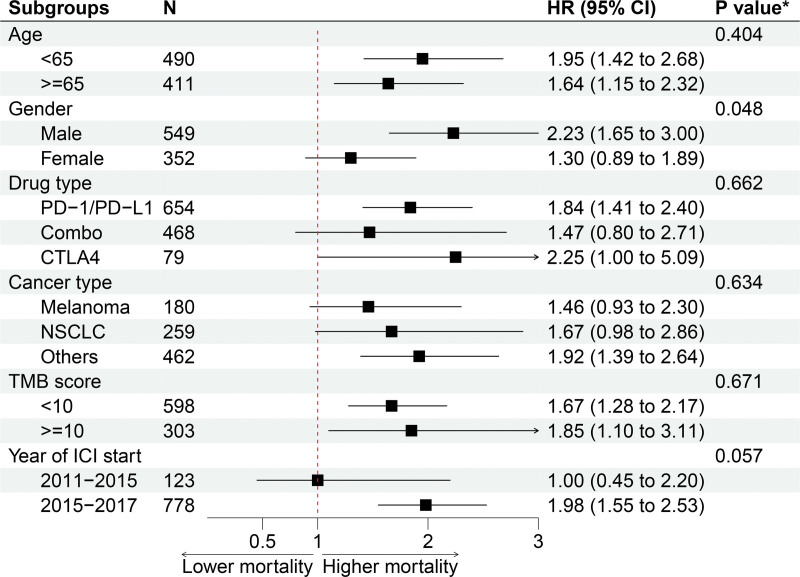
The stratified analysis for unadjusted HR in the subgroups of covariates. * *P* for interaction. HR = hazard ratio.

Stratified analysis by subgroups according to study variables, including gender, age, TMB score, cancer type, type of drug, and start year of ICIs, is shown in Figure 4. When stratified by age, male patients with liver metastases had a 123% higher risk of death than male patients without liver metastases (HR: 2.23, 95% CI: 1.65–3.00), while female patients had only a 30% increased risk (HR: 1.30, 95% CI: 0.89–1.89). The prognostic efficacy of ICIs in patients with liver metastases was significantly different by gender (*P* for interaction < .048).

To further analyze the potential factors affecting the survival of patients treated with ICIs, we performed univariate analysis. We included a total of 5 indicators: gender, age, TMB score, cancer type, and drug type, of which TMB score, cancer type, and drug type were statistically significant (*P < *.05, Table 2). The crude HR for patients with a TMB score ≥ 10 was 0.54 (95% CI: 0.43–0.67), indicating a lower risk of death with higher TMB scores. A statistically significant difference was also observed between NSCLC and melanoma (*P < *.0001), indicating a 62.0% reduction in the risk of death in melanoma patients relative to NSCLC patients. And a significant reduction in the risk of death with CTLA-4 and the combination regimen relative to PD-1/PD-L1 (*P < *.001). Therefore, we concluded that TMB score, cancer type, and drug type might influence the survival prognosis of patients treated with ICIs. Based on these data, we used a Cox proportional hazards model to adjust for confounding factors such as sex, age, TMB score, cancer type, and type of drug. We could still find that HR was 1.67 (95% CI:1.32–2.12) in the liver metastasis group relative to patients without liver metastasis, which was an independent factor affecting the prognosis of patients treated with ICIs (Table 3).

**Table 2:** Univariate analysis for risk factors of overall survival in advanced solid tumors treated with immune checkpoint inhibitors.

	Statistics	HR (95% CI)	*P*-value
Age (yr)			.600
<65	490 (54.4%)	1	
≥65	411 (45.6%)	1.05 (0.87, 1.27)	
Sex			.091
Male	549 (60.9%)	1	
Female	352 (39.1%)	1.18 (0.97,1.42)	
TMB score			<.0001
<10	598 (66.4%)	1	
≥10	303 (33.6%)	0.54 (0.43, 0.67)	
Drug type			
PD-1/PD-L1	654 (72.6%)	1	
Combo	168 (18.6%)	0.62 (0.48, 0.80)	.0003
CTLA-4	79 (8.8%)	0.54 (0.39, 0.75)	.0003
Cancer type			
NSCLC	180 (20.0%)	1	
Melanoma	259 (28.7%)	0.38 (0.29, 0.49)	<.0001
Others	462 (51.3%)	0.81 (0.65, 1.02)	.072

CI = confidence interval, CTLA-4 = cytotoxic T lymphocyte-associated antigen 4, HR = hazard ratio, NSCLC = non-small cell lung cancer, PD1 = programmed cell death protein 1, TMB = tumor mutation burden.

**Table 3:** Liver metastasis and multivariate hazard ratio of overall survival with 95% confidence intervals in advanced solid tumors.

	Event/N	Non-adjusted	Adjust I*	Adjust Ⅱ†
Liver metastasis				
No	362/762	1	1	1
Yes	89/139	1.80 (1.42, 2.27)	1.78 (1.41, 2.25)	1.67 (1.32, 2.12)
After IPTW	451/901	1.69 (1.32, 2.18)	-	-
After PS-matching	139/556	1.71 (1.34, 2.18)	-	-
PS-adjusted	139/556	1.67 (1.31, 2.12)	-	-

CTLA-4 = cytotoxic T lymphocyte-associated antigen 4, IPTW = inverse probability of treatment weighted, NSCLC = non-small cell lung cancer, PD1 = programmed cell death protein 1, PS = propensity score, TMB = tumor mutation burden.

* This model was adjusted of sex, age(<65, ≥65).

† This model was adjusted of sex, age(<65, ≥65), TMB score(<10, ≥10), Cancer type(NSCLC, Melanoma, Others), Drug type(PD-1/PD-L1, Combo, CTLA-4).

### 3.3. Sensitivity analysis

To further assess the sensitivity of the data. We first performed propensity score (PS) matched analysis, adjusted for the number of patients with and without liver metastases, and re-enrolled 139 patients with liver metastases and 556 patients without liver metastases for baseline analysis. We found that there were relative differences in gender, age, TMB score, and cancer type. However, there were still differences in gender and drug type, showing that the differences in baseline characteristics after PS matching remained significant (see Table S1, Supplemental Digital Content, https://links.lww.com/MD/Q754). IPTW eliminated the inherent difference between the 2 groups. Then it showed that patients with liver metastases still had a significantly higher risk of death than patients without liver metastases (HR: 1.69, 95% CI: 1.32–2.18). Further Cox proportional hazards assessment of PS-adjusted data and adjusting for variables identified liver metastases as an independent risk factor for treatment with ICIs (HR:1.67, 95% CI:1.31–2.12).

## 4. Discussion

Advances in cancer therapeutics have established ICIs as a cornerstone of advanced solid tumor management, demonstrating significant clinical efficacy. However, the survival benefits of ICIs remain controversial in patients harboring liver metastases. Clinical evidence consistently indicates inferior outcomes in solid tumor patients with hepatic metastases receiving ICIs.^[3–8,14]^ In this retrospective cohort study, we analyzed 901 advanced solid tumor patients who received ≥ 1 cycle of ICI therapy, stratifying them by hepatic metastasis status. Our findings revealed significantly shorter OS in the liver metastasis cohort compared to non-metastatic counterparts, with this disparity being particularly pronounced in male patients. Multivariate analysis confirmed hepatic metastasis persistence as an independent negative prognostic factor for ICIs efficacy after controlling for confounding variables.

The underlying mechanisms driving ICIs resistance in liver metastasis patients remain incompletely understood. Current hypotheses suggest hepatic metastases may foster immunosuppression through multiple pathways: recruitment of immunosuppressive macrophages, induction of antigen-specific T-cell apoptosis in hepatic microenvironments, and synergy with peripheral immune tolerance mechanisms. Jeffrey Bluestone et al employed a dual-tumor immunocompetent mouse model to demonstrate LM-associated expansion of regulatory T cells and CD11b + myeloid-derived suppressor cells, which collectively suppress effector T-cell function.^[15]^ Using a murine LM model, Weiping Zou et al demonstrated metastasis-driven accumulation of hepatic CD11b + F4/80 + macrophages that promote CD8 + T-cell apoptosis via Fas-FasL pathway activation.^[16]^ Complementary research identified KIAA1199-mediated neutrophil recruitment through the TGFβ-CXCL3/1-CXCR2 axis as another immunosuppressive mechanism.^[17]^ In contrast, the results of the IMpower150 study showed that clinical benefit in OS was observed with atezolizumab, bevacizumab, and combination chemotherapy in the population with LM. The benefit was greater than in patients without LM.^[10]^ This may be associated with improved immune tolerance in LM by immunization combined with targeted therapy, which requires further studies to confirm.

Our study also revealed that male patients with LM had a worse prognosis than that of female patients in the treatment of ICIs. The possible mechanism was gender-induced hormonal differences. Androgen significantly inhibited the proliferation and function of CD8 + T cells by regulating the expression of USP18, while estrogen did not affect CD8 + T cells and the number of circulating CD4 + T cells in females was higher than that in males of the same age.^[18]^

In the clinical problem of poor prognosis of ICIs in patients with LM, previous studies have been small-scale cohort studies. In contrast, this study included a large number (901 patients) and rich types of cancer (including lung cancer, liver cancer, melanoma, colorectal cancer, and most other advanced solid tumors), which can be analyzed and studied in many aspects. On the other hand, the current research on the prognosis of ICIs in patients with advanced solid tumor LM is in the exploratory stage. Through a comprehensive analysis of this large dataset, this paper determines that LM may be independent predictors of poor prognosis of ICIs in patients with advanced solid tumors, providing some research ideas and clinical experience for subsequent mechanistic studies.

However, our study also has some limitations. First, the data in this study were derived from the Memorial Sloan Kettering Cancer Center, which belongs to the single-center data in the United States and may not be generalizable to other regions based on geographical and ethnic differences. Second, this study belongs to a secondary analysis of retrospective data, and the inherent limitations of the original data are inevitable. For example, we lacked relevant information on PFS and could not further analyze the effect of liver metastasis on PFS. Finally, all patients were screened strictly according to the inclusion and exclusion criteria of the clinical trials rather than randomized, contributing to the lack of generalizability to some extent.

In summary, LM appears to be an independent factor associated with a negative prognosis for ICI efficacy in advanced solid tumors, with preliminary evidence suggesting this association may be more pronounced among male patients. Future research should aim to explore sex-specific immune mechanisms more systematically and assess combinatorial approaches to mitigate hepatic immunosuppression, while maintaining a balanced perspective on the potential role of sex as a biological variable in therapeutic responses.

## Acknowledgments

We gratefully thank you for the raw data from Robert M. Samstein and the statistical support from Empower U team of the Department of Epidemiology and Biostatistics, X&Y Solutions Inc. in Boston.

## Author contributions

**Conceptualization:** Shan Lin.

**Data curation:** Shan Lin.

**Formal analysis:** Shan Lin.

**Funding acquisition:** Xiaoruo Yu.

**Methodology:** Shan Lin.

**Supervision:** Xiaoruo Yu.

**Writing – original draft:** Shan Lin, Xiaoruo Yu.

**Writing – review & editing:** Shan Lin, Xiaoruo Yu.

## Supplementary Material


