# Supplementary material for: Prognostic impact of liver metastases on immunotherapy in patients with advanced solid tumors: A secondary analysis of MSK-IMPACT retrospective cohort
Source: Medicine (Baltimore). 2025 Nov 21;104(47):e46137. doi: 10.1097/MD.0000000000046137 (PMC12643660; doi:10.1097/MD.0000000000046137)
Supplement: Supplementary file 1 [file medi-104-e46137-s001.pdf]

Table S1. Baseline characteristics of advanced solid tumors with or without liver metastasis after propensity score matching.

	Liver metastasis		*P*-value
	No	Yes	
N	556	139	
Age at report			0.848
<65	315 (56.7%)	80 (57.6%)	
⩾65	241 (43.3%)	59 (42.4%)	
Mean+SD	60.7 ± 14.0	61.1 ± 13.2	0.775
Sex			<0.001
Male	417 (75.0%)	83 (59.7%)	
Female	139 (25.0%)	56 (40.3%)	
TMB score			1
< 10	416 (74.8%)	104 (74.8%)	
⩾10	140 (25.2%)	35 (25.2%)	
Median (Min-Max)	5.9 (0.0-178.4)	5.6 (0.0-181.8)	0.804
Cancer type			0.327
NSCLC	96 (17.3%)	31 (22.3%)	
Melanoma	119 (21.4%)	31 (22.3%)	
Others	341 (61.3%)	77 (55.4%)	
Drug type			<0.001
PD-1/PDL-1	474 (85.3%)	98 (70.5%)	
Combo	81 (14.6%)	31 (22.3%)	
CTLA4	1 (0.2%)	10 (7.2%)	

Differences are compared using the chi-square test (or Fisher's exact test) for categorical measures and the Kruskal−Wallis test for continuous measures. TMB: Tumor Mutation Burden. NSCLC, Non-Small Cell Lung Cancer. ICI, Immune Checkpoint Inhibitors. CTLA-4, Cytotoxic T Lymphocyte-associated Antigen 4. PD1, Programmed Cell Death Protein 1.

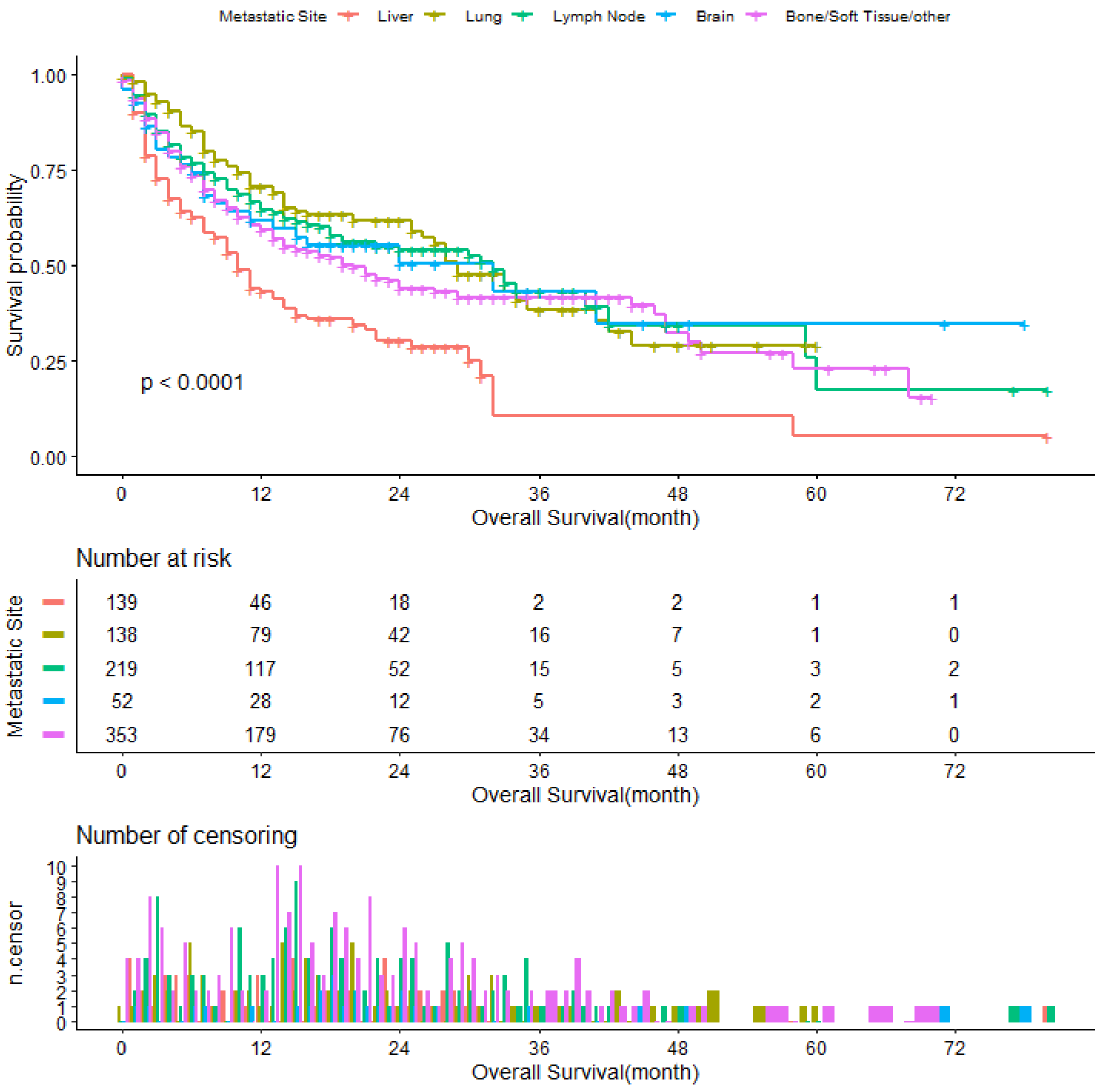

Figure S1. Kaplan-Meier curves of overall survival in advanced solid tumors with various metastasis sites.
